# The Prevalence of Nonalcoholic Fatty Liver Disease and Related Metabolic Comorbidities Was Associated with Age at Onset of Moderate to Severe Plaque Psoriasis: A Cross-Sectional Study

**DOI:** 10.1371/journal.pone.0169952

**Published:** 2017-01-18

**Authors:** Xin Xu, Lina Su, Yunlu Gao, Yangfeng Ding

**Affiliations:** Department of Dermatology, Shanghai Dermatology Hospital, Shanghai, China; Kaohsiung Medical University Chung Ho Memorial Hospital, TAIWAN

## Abstract

Nonalcoholic fatty liver disease (NAFLD) has been found to be highly prevalent in psoriatic patients. Adult onset psoriasis could be divided into either early or late onset psoriasis. The associations between NAFLD and related metabolic comorbidities and age at onset of psoriasis have not yet been investigated. Our study was to evaluate the associations between prevalence of NAFLD and related metabolic conditions and early, late, and childhood onset psoriasis. A cross-sectional observational study was conducted on patients with moderate to severe plaque psoriasis. Data on clinical characteristics of NAFLD and related metabolic diseases (diabetes, hypertriglyceridemia, hyperuricemia, and metabolic syndrome) were collected. The prevalence of NAFLD in 439 patients (mean: 51±14 years, range: 18–85 years) was 55.8%. NAFLD was frequently identified in early onset patients (74.2%), and this diagnosis was particularly common in patients currently younger than 40 (85.3%). Diabetes was the least prevalent component of metabolic syndrome in early onset patients with metabolic syndrome but the most often found component in late onset ones. Patients with childhood onset psoriasis had the lowest frequencies of all metabolic comorbidities except hyperuricemia among the three groups. In the multivariate analyses, early onset was independently and positively associated with NAFLD, hypertriglyceridemia and hyperuricemia and independently and negatively associated with diabetes among early and late onset patients. The results suggested prevalence of NAFLD and related metabolic comorbidities was associated with age at onset of moderate to severe plaque psoriasis. Early onset of psoriasis was independently associated with greater odds of NAFLD, hypertriglyceridemia, hyperuricemia and smaller odds of diabetes compared to late onset. Early onset patients have metabolic syndrome mainly related to lipid disorders and abnormal glucose metabolism was not often involved.

## Introduction

Recently, nonalcoholic fatty liver disease (NAFLD) had been found to be highly prevalent in psoriasis patients, and psoriasis is considered to be an independent risk factor for NAFLD[1,2,3,4,5]. As NAFLD may play a role in the pathogenesis of metabolic syndrome[6], and severity of psoriasis[3,4], may affect treatment options, screening for NAFLD seems more important than before in psoriatic patients management. Data regarding NAFLD in Asian psoriatic patients is lacking.

Recent studies have examined the association between childhood and very late onset psoriasis and metabolic and cardiovascular comorbidities[7,8]. Age at onset was not found to be an independent risk factor for comorbidities in patients with childhood onset psoriasis. In 1985, Henseler and Christophers described two distinct subtypes of adult chronic plaque psoriasis; early-onset psoriasis (EOP), which presents before 40 years of age and late-onset psoriasis (LOP), which first presents after the age of 40 years[9]. Several studies have shown that clinical and genetic features and responses to treatment differ between early and late onset psoriasis[10,11,12]. However, few studies have investigated whether the two types differ in their association with metabolic comorbidities; therefore, we conducted a cross-sectional observational study to evaluate the associations between NAFLD and related metabolic comorbidities and age at onset of moderate to severe psoriatic patients in Shanghai.

## Methods

The wording of the manuscript is suitable for publication.

### Participants and study design

All data involved in the study were from routine examinations of psoriatic patients receiving outpatient and inpatient care at the Shanghai Dermatology Hospital. The data were collected and sorted from the hospital database in which patient data were anonymized. Patients who had their visit from Jan 1, 2014 to Dec 31, 2015 and were diagnosed chronic plaque psoriasis, which was the most common type of psoriasis, were included. We defined patients with moderate to severe psoriasis as those with a Psoriasis Area and Severity Index (PASI) score of more than 10 and with a body surface area (BSA) involvement of more than 10%, and/or receiving systemic treatment (MTX, acitretin, biologics) in our hospital over the past 3 years. As psoriasis severity fluctuates and sometimes hard to evaluate precisely in relatively mild phase, we used systemic treatment as a criterion because systemic treatments were only used in moderate to severe psoriasis in our hospital, following Chinese guidelines for treatment of psoriasis. Data collected for each patient included demographics (age and sex); characteristics of psoriasis (age at onset and history of treatment); and diagnosis of NAFLD or a related metabolic disease (NAFLD, diabetes, hypertriglyceridemia, hyperuricemia, and metabolic syndrome). Data for blood tests conducted at Shanghai Dermatology Hospital were also obtained from the database. Because acitretin may influence blood lipid levels, patients receiving acitretin for at least 1 month within the six months or at least 2 weeks within one month prior to blood testing were excluded.

Diabetes diagnosis was defined as a fasting blood glucose greater than or equal to 6.1 mmol/L or reported use of oral glucose-lowering medication or insulin. Hypertriglyceridemia was defined as serum triglycerides greater than or equal to 1.7 mmol/L. NAFLD was diagnosed using abdominal ultrasonography performed by certified and experienced technicians. Patients with any of the following possible secondary causes of fatty liver disease were excluded: excessive alcohol consumption, hepatitis B or C, and use of oral medication associated with development of fatty liver (corticosteroids) within the past six months. Hyperuricemia was defined as a serum urate level greater than 7.0 mg/dL for men and greater than 6 mg/dL for women. Metabolic syndrome was defined, according to Adult Treatment Panel III criteria, as the presence of at least 3 of the following 5 traits[13]: (1) waist circumference greater than 90 cm in men or greater than 80 cm in women within the Chinese population; (2) serum triglycerides greater than or equal to 1.7 mmol/L or drug treatment for elevated triglycerides; (3) serum high-density lipoprotein cholesterol less than 1.03 mmol/L in men and less than 1.3 mmol/L in women or drug treatment for low high-density lipoprotein cholesterol; (4) blood pressure greater than or equal to 130/85 mmHg or treatment of previously diagnosed hypertension; and (5) fasting plasma glucose greater than or equal to 5.6 mmol/L or drug treatment for elevated blood glucose.

When early onset patients were compared to late onset patients for metabolic comorbidity prevalence in multivariate analysis, age and age at onset were both potential impact factors, which were considered entry into multivariate analysis. But when early onset patients currently under age of 40 years were compared to late onset patients who were all over age of 40 years, age and age at onset were non-independent factors. To investigate whether age at onset was independently associated with metabolic comorbidity prevalence, we compared early onset patients currently over the age of 40 years with late onset patients in multivariate models including age, sex and age at onset. To compare early onset patients currently under the age of 40 years with late onset patients, we entered either age or age at onset into multivariate analyses according to the results obtained from the analysis comparing early onset patients currently over age of 40 years and late onset patients.

### Statistics

Student’s t tests and Chi-square or fisher’s exact tests were used to test for significant differences in the distributions of continuous and categorical data, respectively. Logistic regression analyses were used to evaluate the relationship between age at onset of psoriasis and metabolic comorbidities within multivariate models. Statistical analysis was performed using SPSS 19.0 software (IBM Corp, Armonk, NY).

## Results

Overall, 439 patients (mean age: 51 ±14 years, range 18–85 years) were included in the study, and no patients had missing data. Of the patients, 229(52.2%) had early onset psoriasis, 183(41.7%) had late onset psoriasis, and the remaining 27 had childhood onset psoriasis with an age at onset under 18 years. In our study, the prevalence of NAFLD in psoriasis patients was 55.8% overall, 54.5%for males and 61.3% for females. Early onset patients had a much higher prevalence of NAFLD (74.2%) than other groups (Table 1). In the univariate analyses, significantly higher frequencies of NAFLD, hypertriglyceridemia, hyperuricemia and a lower frequency of diabetes were identified in early onset relative to late onset patients. Early onset patients also had significantly higher frequencies of NAFLD, hypertriglyceridemia and metabolic syndrome compared to childhood onset patients. Hypertriglyceridemia (n = 45,97.8%), excessive waist circumference (n = 42,91.3%) and hypo-HDL (n-29,63.0%) were the three most common metabolic syndrome components in early onset patients with metabolic syndrome, higher fasting plasma glucose was the least common component, and 10 of them (21.7%) had 4 or more components, while higher fasting plasma glucose (n = 23.88.5%), hypertriglyceridemia (n = 21,80.8%) and excessive waist circumference (n = 19,73.1%) were the three most common components among their late onset counterpart and 16 of them (61.5%) had 4 or more components.

**Table 1:** Clinical characteristics and metabolic diseases according to age at onset (univariate analysis).

	EOP^a^	LOP^a^	COP^a^	P-value
Mean age (years), mean ± SD	42.8+-11.1	61.9+-8.6	32.1+_8.1	0.000^b^
				0.009^c^
Sex, male n (%)	198(86.5%)	144(78.7%)	18 (66.7%)	NS^b^
				0.007^c^
Age at onset (years), mean ± SD	27.4+-6.8	53.3+-8.0	13.7+-1.0	NA^b^
				NA^c^
NAFLD, n (%)	170 (74.2%)	74(40.4%)	6(22.2%)	0.000^b^
				0.000^c^
Diabetes, n (%)	21(9.2%)	58(31.7%)	1(3.7%)	0.000^b^
				NS^c^
Hypertriglyceridemia, n (%)	99(43.2%)	47(25.7%)	3(11.1%)	0.009^b^
				0.004^c^
Hyperuricemia, n (%)	43(18.8%)	5(2.7%)	6(22.2%)	0.000^b^
				NS^c^
Metabolic syndrome, n (%)	46(20.1%)	26(14.2%)	1(3.7%)	NS^b^
				0.037^c^
Excessive Waist circumference, n (%)	44(19.2%)	29(15.8%)	1(3.7%)	NS^b^
Hypo-HDL, n (%)	59(25.8%)	46(25.1%)	4(14.8%)	NS^b^
Higher Fasting plasma glucose(> = 5.6mmol/L), n (%)	21(9.2%)	58(31.7%)	1(3.7%)	0.000^b^
Hypertension, n (%)	21(9.2%)	29(15.8%)	1(3.7%)	NS^b^

NS, not significant; NA not applicable; NAFLD, nonalcoholic fatty liver disease

a: early onset psoriasis (EOP), late onset psoriasis (LOP), childhood onset psoriasis (COP).

b: significance level between early onset and late onset psoriasis

c: significance level between early onset and childhood onset psoriasis.

We divided patients into three groups (early onset currently aged 18–39 years, early onset currently aged over 40 years, late onset) to examine patterns in comorbidity frequencies (Table 2). All three groups had similar male/female ratios. NAFLD was identified more frequently in early onset patients currently aged 18–39 (85.3%) than early onset patients currently aged over 40 years and late onset patients. Similar frequencies of other comorbidities were identified in early onset patients currently aged 18–39 and over 40 years. NAFLD, hypertriglyceridemia and hyperuricemia were more frequently identified and diabetes was less frequently identified in early onset patients, regardless of current age, than late onset patients. Metabolic syndrome was more frequently identified in early onset patients age over 40 years than late onset patients.

**Table 2:** Prevalences of metabolic comorbidities in early onset patients currently aged 18–39 years, early onset patients currently aged over 40 years and late onset patients.

	EOP^a^	LOP^a^	P-value
NAFLD			
18-39yrs, (%)	85.3		<0.05^b^^,^^c^
> = 40yrs, (%)	65.4	40.4	
Hypertriglyceridemia			
18-39yrs, (%)	43.4		<0.05^b^^,^^c^
> = 40yrs, (%)	41.5	25.7	
Diabetes			
18-39yrs, (%)	8.5		<0.05^b^^,^^c^
> = 40yrs, (%)	9.2	31.7	
Hyperuricemia			
18-39yrs, (%)	22.5		<0.05^b^^,^^c^
> = 40yrs, (%)	16,9	2.7	
Metabolic syndrome			
18-39yrs, (%)	18.6		NS^b^<0.05^c^
> = 40yrs, (%)	22.3	14.2	

NAFLD, nonalcoholic fatty liver disease.

a: early onset psoriasis (EOP), late onset psoriasis (LOP).

b: significance level between early onset patients currently aged 18–39 years and late onset patients.

c: significance level between early onset patients currently aged over 40 years and late onset patients.

As metabolic syndrome was significantly associated with NAFLD and hyperuricemia and could moderate the association between these outcomes and age at onset, we used metabolic syndrome as a predictor in the multivariate analysis. In our study, early onset patients had longer disease duration than late onset patients on average. Longer disease duration could possibly be associated with higher prevalence of metabolic diseases which may affect the association between metabolic diseases and age at onset So we used disease duration as a predictor when comparing early onset patients aged under 40 years and late onset patients. But when comparing early onset patients aged over 40 years and late onset patients, as disease duration obviously depends on age at onset, we selected age at onset rather than disease duration for analysis. Multivariate analysis showed that in patients with early onset psoriasis currently aged under 40 years, age, sex and metabolic syndrome were not significantly associated with NAFLD or hyperuricemia and age and sex were not significantly associated with hypertriglyceridemia, diabetes or metabolic syndrome. Among early onset patients currently aged over 40 and late onset patients, NAFLD, hypertriglyceridemia and hyperuricemia were identified significantly more frequently and diabetes was identified significantly less frequently in early onset patients (Table 3). Among early onset patients currently aged under 40 and late onset patients, age at onset instead of current age was selected for entry into the multivariate analyses to ensure results consistent with those identified in the comparisons of early onset patients currently aged over 40 and late onset patients as in that comparison, age at onset rather than current age was associated with metabolic comorbidity prevalence. The results suggested that NAFLD, hypertriglyceridemia, and hyperuricemia were also identified significantly more frequently and diabetes was also identified significantly less frequently among early onset patients currently aged under 40 years than late onset patients (Table 4). Integrating the aforementioned results, among early onset and late onset patients, early onset was independently and positively associated with NAFLD, hypertriglyceridemia and hyperuricemia and negatively associated with diabetes.

**Table 3:** Multivariate analysis comparing patients with early onset psoriasis currently aged over 40 years and patients with late onset psoriasis.

	P-value	OR(95%CI)
NAFLD		
Age	0.188	
Sex	0.006	2.382(1.281–4.429)
Age at onset (EOP vs LOP)^a^	0.005	0.434(0.242–0.779)
Metabolic syndrome	0.000	0.267(0.132–0.537)
Hypertriglyceridemia		
Age	NS	
Sex	0.082	
Age at onset (EOP vs LOP)^a^	0.000	0.351(0.196–0.628)
Diabetes		
Age	NS	
Sex	0.001	0.122(0.037–0.408)
Age at onset (EOP vs LOP)^a^	0.000	3.987(1.835–8.661)
Hyperuricemia		
Age	NS	
Sex	NS	
Age at onset (EOP vs LOP)^a^	0.000	0.161(0.049–0.525)
Metabolic syndrome	NS	
Metabolic syndrome		
Age	NS	
Sex	0.020	0.239(0.072–0.798)
Age at onset (EOP vs LOP)^a^	0.111	

OR, odds ratio; CI, confidence interval. NAFLD, nonalcoholic fatty liver disease.

a: early onset psoriasis (EOP), late onset psoriasis (LOP). NS, not significant

**Table 4:** Multivariate analysis comparing patients currently aged under 40years with early onset psoriasis and patients with late onset psoriasis.

	P-value	OR (95%CI)
NAFLD		
Sex	0.002	3.880 (1.862–8.082)
Age at onset, (EOP vs LOP)^a^	0.000	0.098 (0.051–0.190)
Metabolic syndrome	0.000	0.124 (0.048–0.324)
Disease duration	NS	
Hypertriglyceridemia		
Sex	0.077	
Age at onset (EOP vs LOP)^a^	0.001	0.422(0.249–0.716)
Disease duration	0.065	
Diabetes		
Sex	0.142	
Age at onset (EOP vs LOP)^a^	0.000	5.406 (2.539–11.511)
Disease duration	NS	
Metabolic syndrome		
Sex	NS	
Age at onset (EOP vs LOP)^a^	NS	
Disease duration	NS	
Hyperuricemia		
Sex	NS	
Age at onset (EOP vs LOP)^a^	0.000	0.106 (0.038–0.295)
Metabolic syndrome	0.021	0.324(0.124–0.846)
Disease duration	NS	

OR, odds ratio; CI, confidence interval. NAFLD, nonalcoholic fatty liver disease.

a: early onset psoriasis (EOP), late-onset psoriasis (LOP). NS, not significant

Comparing early onset and childhood onset patients, early onset was also independently and positively associated with NAFLD and hypertriglyceridemia (Table 5). Combined with the results obtained in the comparison between early onset and late onset patients, these data suggest that early onset of psoriasis is independently associated with NAFLD and hypertriglyceridemia among moderate to severe plaque psoriatic patients with different ages of onset.

**Table 5:** Multivariate analysis comparing patients with early onset psoriasis and childhood onset psoriasis.

	P-value	OR (95%CI)
NAFLD		
Age	0.000	1.052(1.024–1.081)
Sex	NS	
Age at onset (EOP vs COP)^a^	0.000	0.058(0.020–0.170)
Metabolic syndrome	0.004	0.244(0.093–0.640)
Hypertriglyceridemia		
Age	NS	
Sex	NS	
Age at onset (EOP vs COP)^a^	0.007	0.177(0.050–0.627)
Age	NS	
Sex	0.064	
Age at onset (EOP vs COP)^a^	0.087	
Diabetes		
Age	NS	
Sex	NS	
Age at onset (EOP vs COP)^a^	NS	
Hyperuricemia		
Age	NS	
Sex	0.024	0.184(0.042–0.803)
Age at onset (EOP vs COP)^a^	NS	
Metabolic syndrome	NS	

OR, odds ratio; CI, confidence interval. NAFLD, nonalcoholic fatty liver disease.

a: early onset psoriasis (EOP), childhood onset psoriasis (COP). NS, not significant

## Discussion

Our study evaluated the prevalence of NAFLD and related metabolic conditions in patients with moderate to severe plaque psoriasis. In our study, the prevalence of NAFLD in patients (mean: 51 ±14 years, range 18–85 years) was 55.8% In 2005, a large cross-sectional study including 3175 subjects in Shanghai estimated a NAFLD rate of 20.82% within the general population (mean: 52.4 ±15.1 years, range 16–88 years) [14],. Our study showed a much higher prevalence of NAFLD in psoriasis patients than that identified in the general population of Shanghai. This finding is consistent with the conclusion derived in previous studies that psoriatic patients, especially those with moderate to severe psoriasis, had a greater risk of NAFLD[5].

In our study early onset patients had longer disease duration on average than late onset patients. However, it didn’t affect the association between metabolic diseases and age at onset in our study. In comparison between early onset patients aged under 40 and over 40, patients aged over 40 had longer disease duration (21 vs 8 years), but they showed much lower or similar prevalence of metabolic diseases than those aged under 40 (Table 2), Multivariate analysis also indicated no effect of disease duration on metabolic diseases.

Regarding the association between age at onset and psoriasis, some differences in clinical presentation and genetic background have been identified for different ages of onset. Early onset psoriasis has been reported to be associated with more severe and extensive cutaneous involvement and a recurrent clinical course [10]. However, few studies have reported an association between age at onset of psoriasis and metabolic comorbidities. A cross-sectional study including 2201 adults with psoriasis in France found that childhood onset of psoriasis was not associated with higher frequencies of large waist circumference, obesity, diabetes, dyslipidemia and hypertension; however, these outcomes were associated with increased age[7]. Another similar study investigating very late onset psoriasis (over age of 70) found that very late onset patients tended to have lower frequencies of obesity, diabetes, hypertension, and dyslipidemia than ‘early’ onset patients[8]. In our study, comorbidity prevalence did not increase or decrease with age as indicated in the previous study of psoriatic patients; this may be due to the inclusion of only moderate to severe patients, as metabolic diseases have been found to be more prevalent among these patients and more highly associated with systemic inflammation, these patients may have different distribution patterns of metabolic comorbidities than general patients. As psoriasis, NAFLD and metabolic syndrome display considerable overlap in the occurrence of some slightly elevated proinflammatory marker [15], they may have a synergistic effect on patients’ chronic systemic inflammation that, in turn, make psoriasis more difficult to manage. As early onset patients with moderate to severe psoriasis have been found to have a significantly higher prevalence of NAFLD and tendency towards a higher prevalence of metabolic syndrome, they may suffer from more severe systemic inflammation that may lead to more extensive cutaneous involvement and a recurrent clinical course.

NAFLD is considered a hepatic manifestation of metabolic syndrome. It may predict metabolic syndrome emergence and has been found to be an independent predictor of cardiovascular diseases [16,17,18]. Hyperuricemia may also predict the emergence of metabolic syndrome and has also been associated with cardiovascular diseases[19,20]. As early onset was independently and positively associated with both NAFLD and hyperuricemia, early onset patients may have a greater long term risk of metabolic syndrome or cardiovascular diseases. However in our study, metabolic syndrome was identified a little more frequently in early than late onset patients in univariate analysis, but not with significance. That is probably related to the higher prevalence of diabetes in late onset patients.

In our study, we found that the distribution of metabolic syndrome components was largely uneven in early and late onset patients with metabolic syndrome. Lipid disorders (hypertriglyceridemia, hypo-HDL) and diabetes were the most and least often found metabolic syndrome components respectively in early onset patients. As early onset patients also had higher prevalence of NAFLD, it is indicated that early onset patients may have metabolic disorders mainly related to lipid disorders and abnormal glucose metabolism does not often involved. A previous study about general population with metabolic syndrome showed diabetes was more likely to go with three or more other components rather than two only, in contrast, hypertriglyceridemia, hypo-HDL and increased waist circumference were more likely to be with two other components rather than three or more, suggesting diabetes was associated with more advanced stages of metabolic syndrome[21]. In our study late onset patients with metabolic syndrome mostly had diabetes and lipid disorders, and more of them had four or more components than early onset ones, indicating late onset patients had more complicated metabolic abnormality.

Metabolic comorbidities, except for hyperuricemia, were identified significantly less frequently among patients with childhood onset than other onset groups. In the multivariate analysis, childhood onset was negatively associated with NAFLD and hypertriglyceridemia compared with early onset psoriasis. Metabolic syndrome was identified significantly less frequently among childhood onset patients in univariate analysis; however, this association was not significant in multivariate analysis, which is likely due to sample size. In our study, the sample size of childhood onset patients was much smaller than that of patients in the other two types of psoriasis onset, and the smaller sample size may be due to several factors: 1) lower prevalence of plaque psoriasis in childhood onset patients; and 2) less severe psoriasis in childhood onset patients than early or late onset patients. The aforementioned findings imply that childhood onset patients with moderate to severe psoriasis may have less severe metabolic problems and systemic inflammation than early onset patients; therefore, they may also be able to better manage their psoriasis and have better disease prognosis. However, the prevalence of hyperuricemia implied that there was still some risk of metabolic and cardiovascular disease.

In our study, sex was not a risk factor for NAFLD or hypertriglyceridemia prior to the age of 40 years. This finding is not in accordance with disease patterns identified in the general population, in which males were found to have a sex-specific increased risk of NAFLD prior to the age of 50 years, whereas pre-menopausal women were found to have relatively low rates of NAFLD and hypertriglyceridemia[22]. Thus, younger female patients with moderate to severe psoriasis may not be as well-protected by estrogen as females without psoriasis. They should also be screened for NAFLD and hypertriglyceridemia. After the age of 40 years, female patients have been found to tend to have a higher risk of NAFLD than males, which is similar to patterns observed in the general population [22].

Frequent and severe metabolic comorbidities have been found to be associated with more severe psoriasis. However, as psoriasis is a dynamic and chronic disease and may transit between relatively mild and severe periods, whether early onset patients with moderate to severe psoriasis tend to have worse prognosis in the long-term is difficult to evaluate and needs further research.

In summary, prevalence of NAFLD and related metabolic comorbidities was associated with age at onset among moderate to severe plaque psoriasis patients. Early onset patients, especially those currently under age of 40 years, had significantly higher prevalence of NAFLD than patients with late or childhood onset. Early onset was independently associated with a greater odd of NAFLD, hypertriglyceridemia, hyperuricemia and a smaller odd of diabetes compare to late onset. The distribution of metabolic syndrome components was largely uneven in early and late onset patients with metabolic syndrome Hyperuricemia was the only metabolic comorbidities that occurred more frequently in childhood onset patients than patients with early or late adult onset. Doctors should place an increased emphasis on the distinctive distribution patterns of metabolic disorders in patients with moderate to severe psoriasis, especially those with early onset, to improve their metabolic condition and potentially lead to better psoriasis management.

## Supporting Information

S1 File Ethical Statement (Original version). (PDF)

S1 Table Data used for analysis. Groups (age of onset):1, early onset; 2, late onset; 3, childhood onset; NAFLD: non-alcoholic fatty liver; diabetes, hypertriglyceridemia, NAFLD, hyperuricemia, metabolic syndrome, hypo-HDL, hypertension, waist circumference: 1, yes;2,no.
(XLS)

S1 Text Ethical Statement (English translation). (DOCX)

S2 Text STROBE checklist. (DOCX)
