# Supplementary material for: The Prevalence of Nonalcoholic Fatty Liver Disease and Related Metabolic Comorbidities Was Associated with Age at Onset of Moderate to Severe Plaque Psoriasis: A Cross-Sectional Study
Source: PLoS One. 2017 Jan 18;12(1):e0169952. doi: 10.1371/journal.pone.0169952 (PMC5242531; doi:10.1371/journal.pone.0169952)
Supplement: S1 File — (PDF) [file pone.0169952.s001.pdf]

声明

徐馨医生的文章"Prevalence of Non-alcoholic Fatty Liver Disease and Related Metabolic Comorbidities was Associated with Age of Onset of Moderate to Severe Psoriasis: A Cross-sectional Study"所涉及研究是在医院伦理委员会知晓情况下开展的。因其收集患者过程为常规诊疗过程，收集数据为患者评估过程中必需的常规项目，在医院匿名数据库中调取整理，整个过程中不涉及患者姓名或非常规特殊项目。故我们决定免去其伦理审查程序， 特此声明。

上海市皮肤病医院伦理委员会负责人：

2016-8-4

上海市皮肤病医院 伦理委员会

[illegible]
