# Supplementary material for: The Prevalence of Nonalcoholic Fatty Liver Disease and Related Metabolic Comorbidities Was Associated with Age at Onset of Moderate to Severe Plaque Psoriasis: A Cross-Sectional Study
Source: PLoS One. 2017 Jan 18;12(1):e0169952. doi: 10.1371/journal.pone.0169952 (PMC5242531; doi:10.1371/journal.pone.0169952)
Supplement: S1 Text — (DOCX) [file pone.0169952.s003.docx]

Statement

The research involved in Doctor Xu’s article ‘The Prevalence of Nonalcoholic Fatty Liver Disease and Related Metabolic Comorbidities was associated with Age at Onset of Moderate to Severe Plaque Psoriasis: A Cross-sectional Study’ was conducted with the knowledge of the Ethics Committee of the hospital. The data was from routine examination of patients receiving outpatient and inpatient care in the hospital and collected and sorted from hospital database in which patient data were anonymized. The researchers didn’t have access to patients’ names and other personal information, the research didn’t involve specific examination. Thus we decided to exempt it from requirement of ethical approval.

The Ethics Committee of Shanghai Dermatology Hospital

Director: Zhongjian Chen

Aug 4 2016
